# The *C. elegans* TPR Containing Protein, TRD-1, Regulates Cell Fate Choice in the Developing Germ Line and Epidermis

**DOI:** 10.1371/journal.pone.0114998

**Published:** 2014-12-10

**Authors:** Samantha Hughes, Henry Wilkinson, Sophie P. R. Gilbert, Marcia Kishida, Siyu Serena Ding, Alison Woollard

**Affiliations:** Department of Biochemistry, University of Oxford, Oxford, United Kingdom; East Carolina University, United States of America

## Abstract

Correct cell fate choice is crucial in development. In post-embryonic development of the hermaphroditic *Caenorhabitis elegans*, distinct cell fates must be adopted in two diverse tissues. In the germline, stem cells adopt one of three possible fates: mitotic cell cycle, or gamete formation via meiosis, producing either sperm or oocytes. In the epidermis, the stem cell-like seam cells divide asymmetrically, with the daughters taking on either a proliferative (seam) or differentiated (hypodermal or neuronal) fate. We have isolated a novel conserved *C. elegans* tetratricopeptide repeat containing protein, TRD-1, which is essential for cell fate determination in both the germline and the developing epidermis and has homologs in other species, including humans (TTC27). We show that *trd-1(RNAi)* and mutant animals have fewer seam cells as a result of inappropriate differentiation towards the hypodermal fate. In the germline, *trd-1* RNAi results in a strong masculinization phenotype, as well as defects in the mitosis to meiosis switch. Our data suggests that *trd-1* acts downstream of *tra-2* but upstream of *fem-3* in the germline sex determination pathway, and exhibits a constellation of phenotypes in common with other Mog (*m*asculinization *o*f *g*ermline) mutants. Thus, *trd-1* is a new player in both the somatic and germline cell fate determination machinery, suggestive of a novel molecular connection between the development of these two diverse tissues.

## Introduction

During metazoan development, cells must proliferate in order to generate tissues and organs, but crucially they must adopt the appropriate fate. The control of differentiation is thus absolutely fundamental to the production and maintenance of a correctly functioning organism, with mis-regulation of this process resulting in diseases such as cancer. *Caenorhabditis elegans* provides an excellent model system in which to study cell fate determination due to its almost invariant cell lineage and easily recognizable cell types, thus allowing analysis at single cell resolution [1].

Many cell fate decisions are made early in development, during embryogenesis, however the germline, along with particular neuronal and epidermal cells, are specified later during the larval stages of development. Post-embryonic epidermal lineages involve the lateral seam cells H, V and T, which divide in a re-iterative stem-like manner through a series of asymmetric divisions to produce more seam daughters (self-renewal) as well as those that contribute to the major hypodermal syncytium hyp7 [1]. The asymmetry of these divisions, as well as subsequent cell fate determination, involves molecular pathways conserved throughout the animal kingdom, including Wnt signalling [2], [3] and Runx/CBFβ transcription factor pathways [4], [5], respectively.

In protandric hermaphrodites, germline cells must first proceed from mitosis into meiosis, and subsequently differentiate into either sperm or oocytes. In the mitotic region, self-renewal ensures the maintenance of stocks of germ cells to replenish those that differentiate. Thus the seam cells and germ cells both have stem-like properties, although only the germline stem cells (GSC) have a recognizable niche, regulated by a notch signal emanating from the distal tip cell (DTC) that maintains the mitotic zone [6], [7]. The DTC forms a microenvironment, or plexus, which comprises of a “cap” and long external processes, or cytonemes, which extend into the proximal gonad [8]. As cells move proximally along the germline and away from the DTC, germ cells are no longer under the influence of the niche, and consequentially switch from mitosis to meiosis and begin to differentiate [6].

**Figure 1:**
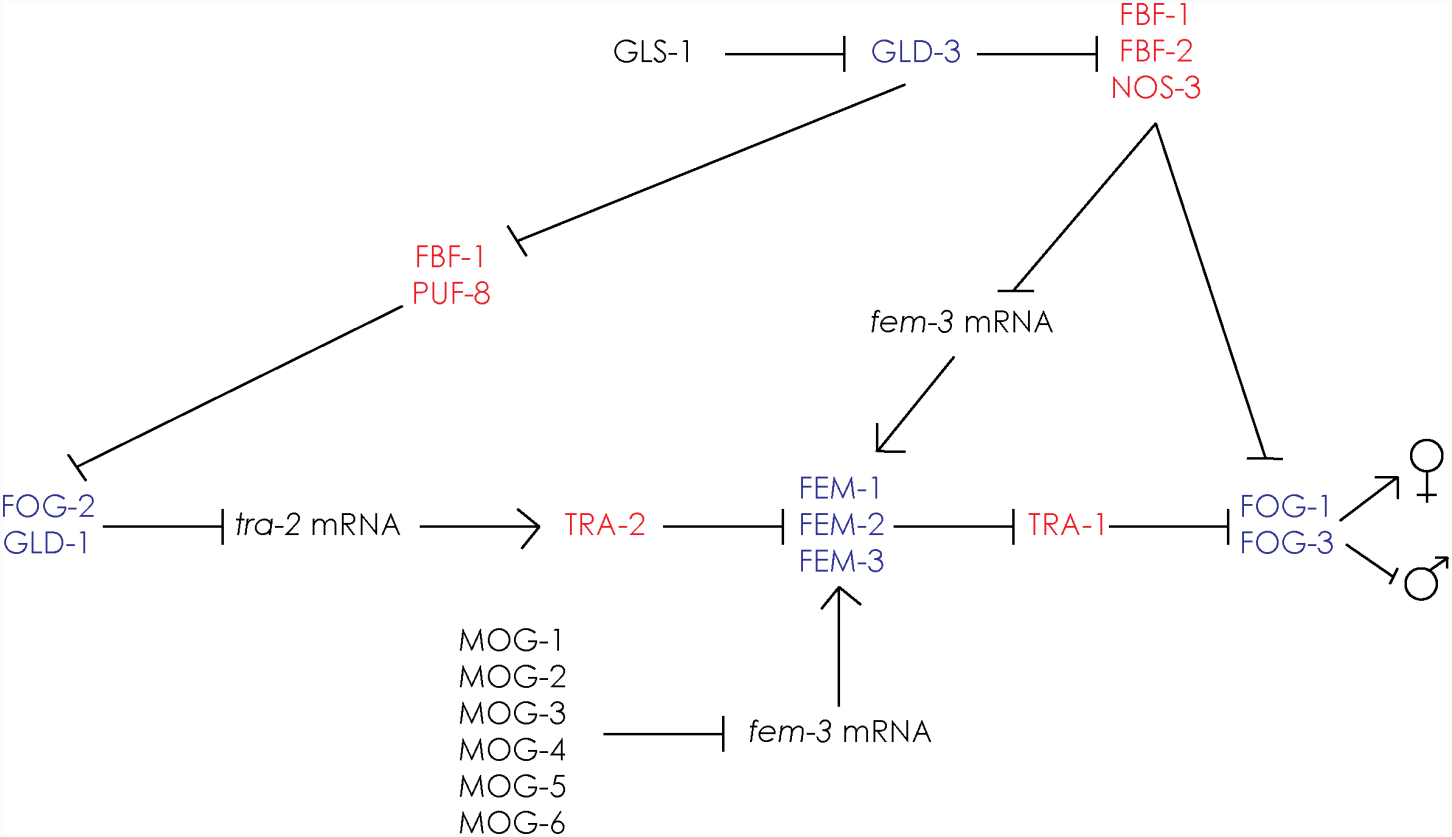
Genetic regulation of germline sex determination. The pathway consists of a cascade of regulatory interactions driving sexual fate. Essentially, *fem-1*, -*2* and -*3* together with *fog-1* and *fog-3* promote spermatogenesis. To allow hermaphrodite animals to switch to oocyte production at the late L4 stage, *tra-2* is repressed by the action of FOG-2 and GLD-1. *fem-3* is repressed at the level of mRNA by multiple factors. Thus, regulation of the balance of TRA-2 and FEM-3 levels allows the timely transition from sperm to oocyte production, in order to generate fully fertile hermaphrodites. Factors that promote male and female fates are highlighted in blue and red, respectively. Adapted from Kimble and Crittenden [14] and Rybarska *et al.* [41].

Hermaphrodites initially produce sperm, switching to oocyte production in late L4 for the remainder of their lives [9], [10]. The switch from spermatogenesis to oogenesis is dependent upon many putative RNA regulatory proteins including FBF-1, FBF-2, NOS-3, GLD-1, 2 and 3, and the MOG family of proteins, as well as the terminal regulators FOG-1 and FOG-3 (Fig. 1) [11]–[15]. Post-transcriptional regulation of germline sex determination makes sense in hermaphrodite animals, where feminizing signals from somatic tissue (set up by the chromosomal X:A ratio) must be transiently over-ridden. Important nodes in the germline sex determination pathway include the masculinizing FEM-3 and the feminizing TRA-2, both of which have been shown to be major targets of the RNA regulatory machinery [13], [16]–[18]. Thus the balance between TRA-2 and FEM-3 activities is an important determinant of whether a germ cell differentiates as sperm or oocyte [19]. This is supported by experimental evidence showing that *fem-3(gf)* single mutants produce only sperm, whereas *tra-2(gf)* single mutants produce only oocytes, whereas the double mutant develops as a fertile hermaphrodite [20]–[22]. Thus, the relative activity of FEM-3 and TRA-2 is the crucial driver of gamete fate. Intriguingly, some of the genes involved in the switch between spermatogenesis and oogenesis in the proximal germline also control the “upstream” decision between mitosis and meiosis in the distal germline, suggesting a possible evolutionary relationship of these regulatory pathways [11].

In this report, we identify a role for the previously uncharacterized gene, *trd-1* (*t*etratricopeptide repeat-containing *r*egulator of *d*ifferentiation), in the regulation of differentiation in both the seam lineages and germline. We observe inappropriate differentiation of normally proliferative seam daughters in the absence of TRD-1, coupled with masculinization of the germline and a defect in the mitosis-meiosis switch. Thus, *trd-1* is required for the correct allocation of cell fate in seemingly disparate lineages.

## Materials and Methods

### Strains and maintenance of worms

Strains were derived from the wild type N2 Bristol strain and maintained at 20°C unless otherwise stated [23]. Strains used are detailed in Table 1. We obtained a single *trd-1* deletion allele (*tm2764*) from the National BioResource Project in Japan. The *tm2764* allele is an 893 bp deletion, removing part of exons 4 and 5. To maintain, and simultaneously outcross, the *trd-1*(*tm2764)* allele, we balanced it with the *hT2*(I;III) reciprocal translocation generating strain *AW626*. Self-progeny of *AW626* included viable and fertile *tm2764/hT2* (essentially wild type animals), *hT2* homozygous animals which are embryonic lethal and *trd-1(tm2764)* homozygous animals that arrest as embryos or larvae, with only a small proportion making it to adulthood. We made a similar strain, *AW912*, which also contained the integrated *scm::gfp* reporter to allow visualization of seam cells in homozygous mutants.

**Table 1:** Strains used in this investigation.

Strain Name	Genotype	Plasmid description
*N2*	Wild type	
*AW60*	*unc-119(e2498)*III*; him-5(e1490); wIs51 [pMF1+ pDP#MM016β*] V	*pMF1[scm::gfp] pDP#MM016β[unc-119(+)]*
*AW626*	*T20B12.1/hT2* (I;III)	*hT2[bli-4(e937) let-?(q782) qIs48]*
*AW912*	*T20B12.1/hT2* (I;III); *him-5(e1490)*; *wIs51[pMF1+ pDP#MM016β]* V	*hT2[bli-4(e937) let-?(q782) qIs48] pMF1[scm::gfp] pDP#MM016β[unc-119(+)]*
*AW1015*	*unc-119(ed3)*III *ouIs10 [pAW584+pAW516+pAW561+unc-119(+)]*	*pAW584[scm::dTtomato] pAW516[dpy-7p::2xNLS::yfp] pAW561[Pwrt-2::gfp-PH]*
*CB3778*	*tra-2(e2020)*II	
*CB4034*	*fem-3(e2006ts) him-8(e1489)*IV	
*CB5098*	*fem-3(q20ts)*IV	
*JK560*	*fog-1(q253ts)*I	
*JK2868*	*unc-119(ed3)*III*; qIs56 *V	*qIs56[lag-2p::GFP + unc-119(+)*]
*JK3101*	*fbf-2(q738)*II	
*JK3354*	*gld-3(q741)/mIn1[mIs14 dpy-10(e218)]* II	*mIs14[myo-2::gfp; pes-10::gfp]*
*JR667*	*unc-119(e2498*)III; *wIs51[pMF1+ pDP#MM016β]* V	*pMF1[scm::gfp] pDP#MM016β[unc-119(+)]*
*NK624*	*unc-119(ed4*)III; *qyIs100[T20B12.1::gfp + pDP#MM016β]*	*pDP#MM016β[unc-119(+)]*
*T20B12.1*	*T20B12.1(tm2764*/+)III	

### Microscopy

DIC (Nomarski) and fluorescent imaging was carried out using a Zeiss AxioSKOP2 microscope with a Zeiss AxioCamMR digital camera. Photomicrographs were taken using a x63 oil immersion objective (Zeiss) and Axiovision software (Release 4.5). Confocal images were taken on a Leica TCS SP5II using Leica Application Suite Advanced Fluorescence Lite software (Release 2.2.1). In both cases, animals were mounted on agarose pads (2% agarose, 0.5% 1-phenoxy-2-propanol in M9) in 0.2% 1-phenoxy-2-propanol.

### Lineage analysis

Lineage analysis was performed as previously described [1]. Seam nuclei were identified based on their morphology, and confirmed where necessary by monitoring expression of the seam specific GFP reporter, *scm::gfp*. Microscopy was performed with Normarski (DIC) optics and an x100 oil immersion objective (Zeiss) using a Zeiss AxioPlan microscope. A total of 7 animals were followed from hatching through the L1 division to the mid-L2 stage.

### RNAi experiments

RNA interference experiments were performed using the Ahringer RNAi library and protocol at 20°C, unless otherwise stated [24]. For seam cell and gonad morphology analysis, dsRNA was delivered by feeding to L4 stage animals and the phenotypes observed in next generation L4 and adults (L4+1 day). For experiments involving temperature sensitive (*ts*) mutants, gravid hermaphrodites were age synchronized by bleaching. To achieve synchronization, gravid animals were washed off plates with M9 buffer into a 15 mL centrifuge tube and pelleted at 2500 rpm for 2 minutes. The supernatant was removed and 6 mL of M9 added followed by 6 mL of bleach solution (12.5 mL 4 M NaOH, 20 mL 12% sodium hypochlorite and 17.5 mL water). Tubes were vigorously shaken for 3 minutes before centrifugation at 2500 rpm for 90 seconds. The supernatant was removed and the egg pellet washed 3 times with M9 buffer. Following the last wash, 10 mL M9 was added and the tubes left rotating at 15°C. Eggs were left to hatch and larvae allowed to develop to L1 in M9 overnight (in the absence of food) at 15°C before being placed on *trd-1* RNAi feeding plates. The plates were then shifted to 25°C, the restrictive temperature, before analysis at the L4 and adult (L4+1 day) stage. Control animals were subjected to identical manipulations.

### Whole worm DAPI

Worms were washed off RNAi plates with M9 buffer and centrifuged for 90 seconds at 2500 rpm in 15 mL centrifuge tubes. The supernatant was removed and the pellet washed with M9 buffer a further 3 times. The worm pellet was then incubated in 150 nM DAPI (4′,6-diamidino-2-phenylindole) in 100% ethanol (200 µL) in a watch glass. After 20 minutes, when the liquid had almost completely evaporated, the nematodes were rehydrated in 1 mL of M9 buffer for 40 minutes, transferred into a micro-centrifuge tube and spun for 60 seconds at 2500 rpm. Worms from the pellet were resuspended in a small volume (5–10 µL) of the remaining supernatant and 3.5 µL of the suspension mounted onto an agarose pad (2% agarose in water) with 2 µL anti-fade (AF3 in PBS, CitiFluor) and sealed with varnish.

### Determination of the mitotic zone

The mitotic zone was determined as previously described [6], [8]. Briefly, the number of germ cell diameters along the distal-proximal axis from the distal tip to the transition zone was counted. The transition zone is defined as being the point at which cells have the crescent shaped nuclei, characteristic of meiotic prophase I, as determined by DAPI staining.

### Western blot analysis

Protein lysates were prepared by boiling 10 adult worms at 95°C for 10 minutes in 20 µL of Laemli Buffer (1.25 mL 0.5 M Tris-HCl pH 6.8, 2.5 mL Glycerol, 2 mL 10% (w/v) SDS, 0.5 mL β-mercaptoethanol and Bromophenol Blue, made up to 10 mL with ddH_2_O). The sample was then centrifuged at 13000 rpm for 10 minutes and the supernatant transferred to a fresh tube. Benzonaze enzyme (0.3 µL) was added and lysates run on a 10% NuPage gel (Novex). The proteins were probed with either a mouse α-MSP (major sperm protein, 4A5, Developmental Studies Hybridoma Bank, University of Iowa) at 1∶1000 dilution or a rabbit α-actin (polyclonal goat α-rabbit HRP, Dako) at 1∶1000. The signal was detected with GE Healthcare ECL reagent and blots were visualized on an autoradiography film.

## Results

### *trd-1* is required for cell fate determination in seam daughters

*trd-1* was initially identified through a genome wide RNAi screen for *C. elegans* genes involved in regulating the number of seam cells [25]. TRD-1 (original gene designation *T20B12.1*) contains six tetratricopeptide repeat (TPR) domains with a characteristic conserved 34 amino acid motif [26] located at the C terminus. TRD-1 has a high level of conservation with mouse and human tetratricopeptide repeat protein 27, TTC27 (E-values of 8e-99 and 1e-95, respectively).

**Figure 2:**
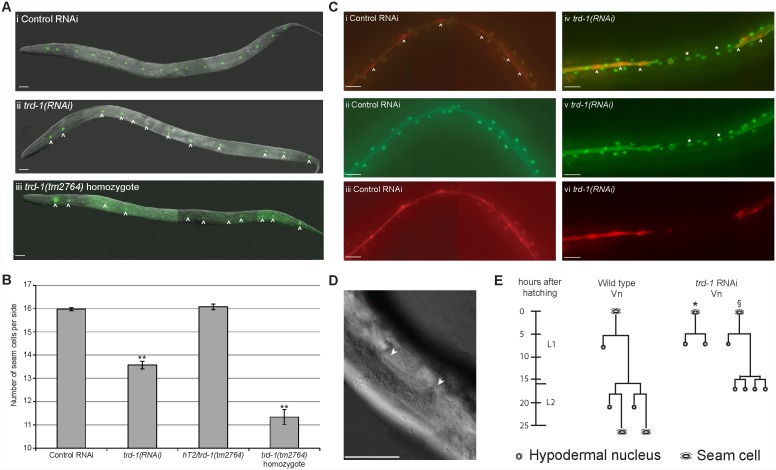
TRD-1 regulates cell fate choice in seam lineages. (A) (i) Wild type late L4 animal carrying the integrated seam cell marker, *scm::gfp* (strain, *JR667*), has 16 seam cells per side. (ii) *trd-1(RNAi*) animals have a reduction in the number of seam cells. Seam cells are indicated by white arrowheads. (iii) *trd-1(tm2764)* homozygous mutants (strain, *AW912*) have a significant reduction in seam cell number. Scale bar, 20 µm. Anterior is to the left and dorsal up in all images. (B) Graph showing average seam cell number. Wild type animals were exposed to empty RNAi feeding vector as a control. *n*>100 for both control and *trd-1(RNAi)* animals. Heterozygotes of genotype *hT2*/*trd-1(tm2764)* have normal numbers of seam cells (*n* = 61), however, *trd-1(tm2764)* homozygotes have a significant reduction in seam cell number to 11.3 (*n* = 45). Error bars represent s.e.m. and ** indicate the 2-sample t-test where each strain was compared to the wild type, where *p*<0.01. (C) (i–iii) L4 Animal (strain, *AW1015*) carrying an integrated seam cell nuclear marker (s*cm::tdTomato*) along with the pleckstrin homology domain *PH::gfp* outlining seam cells and a hyp7 nuclear marker *dpy-7p::yfp*. White arrowheads indicate seam cell nuclei. (iv–vi) *trd-1(RNAi)* animals carrying the same set of markers. There are obvious gaps in the seam cells, where the seam cell fate has been transformed to hypodermal. Asterisks indicate nuclei that inappropriately express *dpy-7::yfp* instead of *scm::tdTomato*. Note that these cells have lost their *PH::gfp* outline, also indicative of transformation towards the hypodermal fate. ii and v are images of the GFP/YFP channel. iii and vi is the red channel to show the tdTomato in the same animal. i and iv are merged imaged of both GFP/YFP and tdTomato channels. All scale bar, 20 µm. Anterior is to the left and dorsal up in all images. (D) Representative image of a trd-1(RNAi) animal displaying shallow or absent alae. Scale bar, 40 µm (E) Representative lineage trace showing the Vn lineage from hatching to late L2. In wild type animals at the L1 stage, Vn divides asymmetrically with the anterior daughter (Vn.a) adopting the hypodermal fate and the posterior daughter (Vn.p) retaining the proliferative fate. Vn.p will divide symmetrically during early L2 followed closely by a further asymmetric division. Vn.pap and Vn.ppp retain the ability to self-renew and will divide again at L3 and L4. We lineaged 7 *trd-1(RNAi*) animals, of which 5 had symmetrized the L1 asymmetric division of V2 (asterisk). In animals that divided in the wild type pattern at the L1 division (2 animals), the L2 asymmetric divisions were again symmetrized towards the hypodermal fate (§). Similar cell fate transformation events were observed in the other V lineages, albeit at lower frequency.

We confirmed a significant reduction in seam cell number from 16 per side in wild type animals to around 13 in *trd-1(RNAi*) animals, *p*<0.01 (Fig. 2A, B). All animals hatched with 10 seam cells per side (data not shown), suggesting no embryonic defects in seam cell identity, prior to the point at which they start dividing. In males, *trd-1* RNAi resulted in male tail abnormalities, including a significant reduction in the number of sensory rays from 9 rays in wild type to an average of 4 in *trd-1(RNAi*) animals, *p*<0.01, which are known to be derived from posterior seam lineages (S1 Figure). In addition, we observed a low penetrance molting defect in *trd-1(RNAi)* animals (S1 Figure), another phenotype that may be associated with seam cell defects [27]. RNAi knockdown was confirmed by quantitative real-time PCR (data not shown). Homozygous *trd-1* mutants derived from the balanced strain *AW912* also displayed a significant reduction in seam cell number in animals that made it through to adulthood, with an average of 11 seam cells per side, *p*<0.01 (Fig. 2A, B). The significant lethality of *trd-1(tm2764)* homozygous mutants precluded detailed analysis of the role of *trd-1* in development in these animals, thus further analysis was performed on *trd-1(RNAi)* animals, in which the lethality was less penetrant.

In wild type animals, asymmetric seam cell divisions at each larval stage produce an anterior daughter that generally differentiates by adopting the hypodermal fate, quitting the cell cycle and fusing with the hyp7 syncytium, and a posterior daughter that maintains the proliferative (seam) fate. Given the decrease in seam cell number observed in *trd-1(RNAi*) animals, we next tested for possible defects in cell fate determination. We performed *trd-1* RNAi on a strain containing both the *scm::dTtomato* and *dpy-7*p*::yfp* reporters as markers of the seam and hypodermal fates, respectively, and observed inappropriate expression of *dpy-7*p*::yfp* in posterior daughters that would normally be expected to maintain the seam fate. These cells had switched off *scm::dTtomato* expression (Fig. 2C). Indeed, the gaps where there was no observed seam cells corresponded to a break in the alae (Fig. 2D).

Lineage analysis was used to confirm this fate change. In *trd-1(RNAi*) animals, asymmetric divisions of V cells were consistently symmetrized to the hypodermal fate, with posterior daughters consequently losing the ability to proliferate further (Fig. 2E). This defect was observed at both the L1 and L2 asymmetric divisions (lineage analysis was not pursued beyond L2). Consistent with this, we observed a reduction in the number of hyp7 nuclei in L4 *trd-1(RNAi)* animals (data not shown), as posterior daughters that would normally contribute nuclei to the hyp7 syncytium during later asymmetric divisions were precluded from further division.

### *trd-1* is expressed in the seam cells and gonad

**Figure 3:**
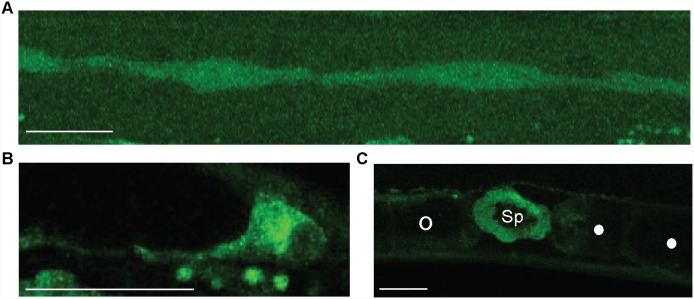
Localization of TRD-1. (A) Strong cytoplasmic expression of *trd-1::gfp* (strain, *NK624*) was observed in seam cells throughout postembryonic development. (B) There is significant expression of *trd-1::gfp* in the distal tip cell (DTC) at L4. (C) Strong expression of *trd-1::gfp* is observed in the spermatheca in young adults. Open circles indicate an unfertilized oocyte and closed circles are fertilized eggs. In all cases, Anterior is to the left, ventral bottom. Scale bars are 20 µm.

Using an integrated GFP reporter (strain *NK624*, a kind gift from David Sherwood [28]), we observed the expression pattern of *trd-1.* Fluorescence microscopy of *trd-1::gfp* shows a strong expression pattern of *trd-1* in the cytoplasm of seam cells (Fig. 3A). In addition, *trd-1* expression was observed in the developing gonad, including strong expression in the DTC (Figure 3B) and in the spermatheca from the late L3 stage onwards (Fig. 3C). Additionally, we observed expression of *trd-1::gfp* in the Pn.p cells and anchor cell, as previously reported [28].

### *trd-1* RNAi results in masculinization of the germline

**Figure 4:**
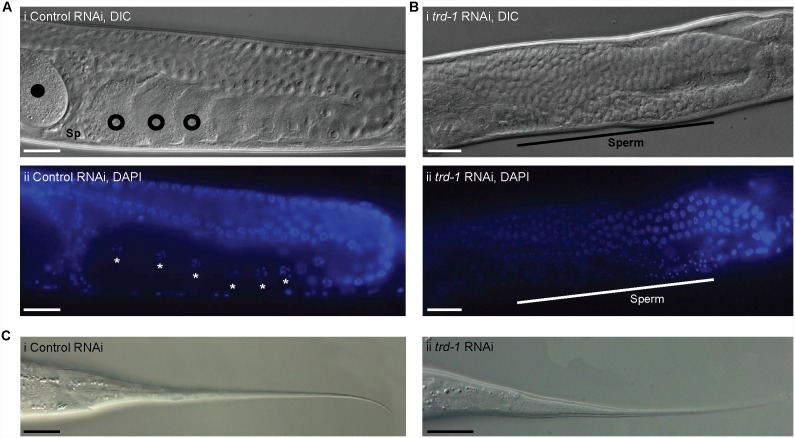
*trd-1* RNAi results in a masculinized germline. (A) A representative (i) DIC and (ii) DAPI image of one proximal gonad arm in a wild type animal. The pachytene region and the presence of oocytes in diakinesis (open circles) are clearly observed in young adults. The condensed chromosomes are clearly visible in the DAPI image (asterisks). Oocytes are pushed through the spermatheca (Sp) to become fertilized embryos (closed circles). (B) Representative (i) DIC and (ii) DAPI images of a young adult animal exposed to *trd-1* RNAi. A masculinized germline, or Mog phenotype, is clearly observed. No oocytes are seen, however there is an extended region of on-going sperm production (black line) containing DAPI staining characteristic of sperm (white line). In all images anterior is to the left and dorsal to the bottom. Scale bar, 20 µm. (C) (i) Wild type whip-like tail tip. (ii) Animals exposed to *trd-1* RNAi also have whip-like tail tips, suggesting no somatic sexual transformation. Scale bar, 20 µm. Anterior is to the left and dorsal up in all panels.

Given the strong expression of *trd-1::gfp* we observed in the gonad, we investigated possible roles for *trd-1* in germline development. The most striking phenotype observed in *trd-1(RNAi)* animals was a significant increase in the amount of sperm and absence of oocytes. DAPI staining of wild type adult (L4+1 day) animals showed developing oocytes in the proximal gonad, which pass through the spermatheca to be fertilized (Fig. 4A). In contrast, in approximately 80% of *trd-1(RNAi*) gonads (*n* = >100) we observed a complete absence of oocytes with a vast increase in the number of sperm (Fig. 4B). Western blotting confirmed that in *trd-1(RNAi)* animals, there is a greater amount of sperm present compared to wild type (S2 Figure). Indeed, the MSP levels of *trd-1(RNAi*) animals are elevated, although not to the same extent as the completely masculinized *fem-3(gf*) mutant (S2 Figure). In contrast to the severe germline masculinization of *trd-1(RNAi*) animals, no somatic masculinization was evident. The hermaphrodite tail of both control and *trd-1* RNAi animals has the simple whip-like structure characteristic of female somatic development (Fig. 4C). We conclude that removal of *trd-1* results in a masculinization of germline, or Mog, phenotype, strongly indicative of a role for *trd-1* in regulating the spermatogenesis-oogenesis switch, promoting oogenesis.

### *trd-1* functions upstream of FEM-3 and FOG-1 but downstream of TRA-2

Germline sex determination is a well characterized but complex pathway (Fig. 1) and disruption of genes within this pathway results in defects in the switch from spermatogenesis to oogenesis. In order to place *trd-1* in the germline sex determination pathway we constructed double mutants with alleles known to affect key regulatory points in the pathway. *fog-1* appears to act as a terminal regulator in the pathway, promoting spermatogenesis, thus mutants produce only oocytes [14], [15], [20]. Knocking down *trd-1* in a *fog-1(lf)* mutant background failed to reverse the Fem phenotype (Table 2), suggesting that *trd-1* acts upstream of *fog-1*.

**Table 2:** Epistasis analysis suggests that *trd-1* functions downstream of *tra-2* and upstream of *fem-3*.

Strain	Temp (°C)	*n*	Sp + Oo (%)	Sp (%)	Oo (%)
control RNAi	20	86	100	0	0
*trd-1(RNAi)*	20	88	20	80	0
*fbf-2(q738)*	20	78	77	0	23
*fbf-2(q738); trd-1(RNAi)*	20	33	18	82	0
*tra-2(e2020, gf)*	20	53	0	0	100
*tra-2(gf); trd-1(RNAi)*	20	52	6	77	17
control RNAi	25	118	100	0	0
*trd-1(RNAi)*	25	123	21	79	0
*fog-1(q253, lf)*	25	65	0	0	100
*fog-1(lf); trd-1(RNAi)*	25	64	0	0	100
*fem-3(e2006, lf)*	25	50	0	0	100
*fem-3(lf); trd-1(RNAi)*	25	50	0	0	100

Worms were bleach synchronized at 15°C then L1s hatched, fed and shifted to 20°C or 25°C as indicated. Following whole worm DAPI on L4+1 day old animals, the presence of sperm (Sp) and oocytes (Oo) was recorded. *fog-1(q253, lf)* and *fem-3(e2006, lf)* mutants produce only oocytes at the restrictive temperature of 25°C, a phenotype which is unaltered by *trd-1* RNAi, suggesting that *trd-1* is operating upstream. The *fbf-2(q738)* mutation has a 23% penetrant feminized germline phenotype at 20°C which is completely suppressed by *trd-1* RNAi. These animals are masculinized to the same extent as *trd-1* RNAi animals alone, suggesting that *trd-1* operates downstream of (or in parallel to) *fbf-2*. *tra-2 (e2020, gf)* mutants are feminized at 20°C, a phenotype that is significantly suppressed by *trd-1* RNAi (again, these animals are masculinized to a similar extent to *trd-1* RNAi animals alone). This suggests that *trd-1* is downstream of *tra-2*. Overall, therefore, *trd-1* appears to be operating downstream of *tra-2* but upstream of *fem-3* to regulate germline sex determination.

Next, we tested *fbf-2*, encoding a PUF (PUmilio and FBF) protein known, in conjunction with *fbf-1,* to inhibit translation of various mRNA targets including *fem-3* and *fog-1* by binding to their 3′UTRs [16]. An *fbf-2(q738*) mutation gives a low penetrance feminized germline phenotype, consistent with previous reports [29], [30], with 23% of animals producing oocytes only (Table 2). We found that RNAi of *trd-1* in this *fbf-2* allele completely suppressed this phenotype, resulting in a highly penetrant masculinized germline (82%), similar to knocking down *trd-1* in a wild type background (Table 2). This suggests that *trd-1* is likely to act downstream of, or in parallel to, *fbf-2*.

**Figure 5:**
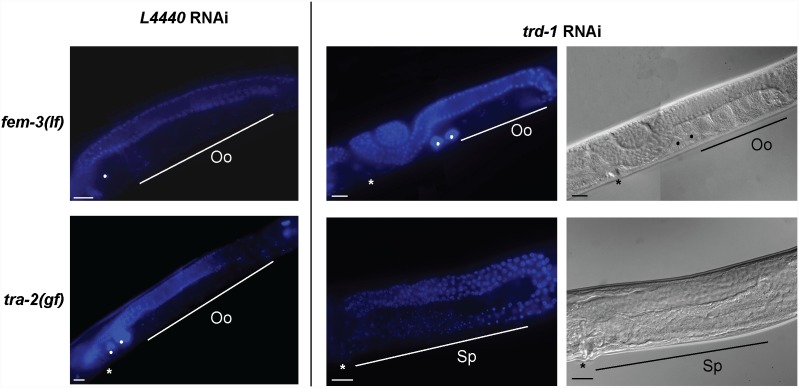
*trd-1* functions downstream of *tra-2* and upstream of *fem-3*. Whole worm DAPI images were taken of young adults (L4+1 day) in the presence and absence of *trd-1* RNAi. The top panel shows that the germlines of *fem-3(lf*) (strain, *CB4034*) mutants are normally feminized at the restrictive temperature of 25°C. *trd-1* (RNAi) does not suppress this phenotype. In contrast, the feminized germline of *tra-2*(*gf*) animals (strain, *CB3778*) is significantly suppressed following *trd-1* RNAi, with most germlines being masculinized. Sp indicates the region of post-meiotic sperm and Oo represents oocytes in diakinesis. Closed circles indicate unfertilized oocytes and the asterisk indicates the location of the vulva. Scale bar, 20 µm. Anterior is to the left and dorsal is up in all images.

The next obvious mutants to test were *fem-3* and *tra-2*, whose activities need to be finely balanced in order to correctly regulate the switch between sperm and oocyte production. In addition, gain of function alleles are available for both *fem-3* and *tra-2*, which makes epistasis experiments easier to interpret. *fem-3(gf)* mutants are masculinized, whereas *tra-2(gf)* mutants are feminized, but double *fem-3(gf); tra-2(gf)* mutants are fully fertile. In the case of *tra-2(gf)* mutants, RNAi of *trd-1* suppressed the Fem phenotype in the majority of animals, giving the masculinized phenotype characteristic of *trd-1* RNAi (Fig. 5, Table 2). Thus, *trd-1* is likely to function downstream of *tra-2*. However, *trd-1* RNAi did not suppress the feminization of *fem-3(lf)* mutants (Fig. 5), suggestive of *trd-1* operating upstream of *fem-3*. Although *trd-1* RNAi made no difference to *fem-3(gf)* mutants at the restrictive temperature (complete masculinization), we did notice a synergistic masculinization at the permissive temperature of 15°C. At this temperature *fem-3(gf)* mutants show only a very weakly penetrant masculinization phenotype (2%), which is enhanced to >90% in the absence of *trd-1* (Table 3). Taken together, these data suggest that *trd-1* is likely to act at the level of *fem-3* in the germline sex determination pathway.

**Table 3:** *trd-1* RNAi enhances the masculinization of *fem-3(gf)* animals at the permissive temperature.

Genotype	Temp (°C)	*n*	Sp + Oo (%)	Sp (%)	Oo (%)
Control RNAi	15	83	100	0	0
*trd-1(RNAi)*	15	72	17	83	0
*fem-3(gf)*	15	56	98	2	0
*fem-3(gf); trd-1(RNAi)*	15	55	7	93	0
Control RNAi	25	52	100	0	0
*trd-1(RNAi)*	25	59	22	78	0
*fem-3(gf)*	25	60	0	100	0
*fem-3(gf); trd-1(RNAi)*	25	50	0	100	0

At the permissive temperature of 15°C, *fem-3(q20, gf*) animals have a very low penetrance Mog phenotype (2%). *fem-3(gf); trd-1(RNAi*) animals display almost complete masculinization of the germline at this temperature, greater than *trd-1* RNAi alone. Thus *trd-1* exhibits a synergistic interaction with *fem-3* at the permissive temperature.

### Role of *trd-1* in the decision between mitosis and meiosis

Several factors known to be important in regulating the sperm-oocyte switch are also involved in the switch from mitosis to meiosis in the distal germ line (Reviewed in [13]). This, coupled with the expression of *trd-1* we observed in the DTC, prompted us to investigate the distal region in more detail, firstly by examining its morphology in *trd-1(RNAi)* animals using a *lag-2*::GFP reporter. During early larval development in wild type animals, the DTC plexus has been reported to comprise of a “cap” with a number of short intercalating processes [8]. At adulthood, the morphology changes, with an increase in the number of short intercalating processes and the formation of long external processes (cytonemes) that extend proximally into the germline [8]. Intriguingly, we found that the cytonemes are formed earlier in *lag-2::gfp; trd-1(RNAi*) animals and are more extensive, being much longer, thicker and more branched compared to wild type (S3 Figure). We found that just 2 wild type animals (*n* = 45) in contrast to all of *trd-1(RNAi*) animals (*n* = 36) displayed abnormal cytonemes.

We next tested for a defect in the mitosis/meiosis switch using two approaches. Firstly, we looked at the size of the mitotic region, confirming that wild type animals had a mitotic zone of approximately 20 cell diameters, as previously reported [8], [11]. This was unchanged in *trd-1(RNAi)* animals (data not shown), suggesting no obvious defect when *trd-1* was silenced in otherwise wild type animals. The second approach was to look for the presence of tumorous germlines (Tum phenotype) indicative of excessive mitosis. We used a sensitized *gld-3* allele (*q741)* in which a low penetrance Tum phenotype is observed, similar to other *gld-3* alleles and *gld-3* RNAi [11], [31]. Gonad tumors were never observed in wild type *trd-1(RNAi*) animals, however a very highly penetrant Tum phenotype was observed in *gld-3(q741); trd-1(RNAi)* animals (Table 4). Tumorous germlines displayed no evidence of meiosis and sperm/oocytes were never detected.

**Table 4:** Germline tumor formation in *trd-1(RNAi)* animals suggests a defect in the mitosis-meiosis switch.

Strain	*n*	Sp + Oo (%)	Sp (%)	Oo (%)	Tum (%)
Control RNAi	33	100	0	0	0
*trd-1(RNAi)*	32	20	59	0	0
*gld-3(q741)*	82	67	0	0	33
*gld-3(q741); trd-1(RNAi)*	44	9	0	0	91

Young adult animals (L4+1day) were stained with DAPI and the gonads scored for the presence of sperm and oocytes (essentially wild type gonads), germlines which contained only sperm (Sp), or only oocytes (Oo) or were tumorous (Tum), a phenotype in which no differentiated nuclei are observed in the germline and all nuclei remain in mitosis. *trd-1* RNAi synergistically enhances the Tum phenotype of *gld-3(q741)* mutants.

## Discussion

Here, we have characterized a novel TPR containing protein, TRD-1, which has important roles in cell fate determination in at least two distinct *C. elegans* tissues. TRD-1 is one of more than 80 TPR containing proteins in *C. elegans*, most of which are uncharacterized [32]. Indeed, although homologs of *trd-1* have been identified in mouse and humans (TTC27), very little is known of their function [32], [33]. In *C. elegans*, TPR containing proteins are slowly being identified, including *sgt-1*, which has been shown to have a potential role in human disease, interacting with human β-amyloid [34]. Thus, *trd-1* is a member of a poorly understood but expanding collection of TPR containing proteins, which might have significant functions in a variety of tissues and biological systems [26], [33].

In seam cell divisions, *trd-1* is required for the maintenance of the proliferative fate, as demonstrated by lineage analysis of *trd-1(RNAi)* animals, and the inappropriate expression of hypodermal-specific markers. Inappropriate cell fate determination was also observed in the germline, with *trd-1(RNAi)* animals displaying a strong masculinization, or Mog, phenotype. In fact, *trd-1*-associated phenotypes bear comparison with the Mog phenotypes associated with mutations in *mog-1-6* in a number of ways, including on-going spermatogenesis during adulthood, synergistic interaction with masculinizing sex determination mutants, maternal effect lethality, molting defects and defects in regulating the mitosis-meiosis switch [31], [35]–[37]. In addition, *trd-1*, like the Mog genes *mog-1-6*, appears to act in the germline sex determination pathway at the level of *fem-3* regulation [12], [17]. Mog genes are required for the repression of *fem-3* via its 3′UTR [17], and thus have a loss-of-function masculinization phenotype similar to that of *fem-3(gf*) animals [12], [16], [17], [35]. Indeed *trd-1(RNAi*) animals also display a Mog phenotype which is comparable to that observed in *fem-3(gf*) worms. This, coupled with our epistasis data placing *trd-1* upstream of *fem-3* but downstream of *tra-2,* strongly suggests that *trd-1* is a component of the *fem-3* regulatory machinery, but it is unclear at present what the biochemistry of this regulation may be. On the molecular level, Mog genes encode likely RNA regulators, but most Mog proteins cloned to date are related to splicing factors [38], suggesting that regulation of the *fem-3* 3′UTR may be indirect. The cytoplasmic localization pattern of *trd-1* we observe is consistent with a role in RNA regulation, but TPR containing proteins have been postulated to have an extremely wide variety of biological roles [26], [33], [39]. Of interest in the context of germline sex determination is the postulated structural similarity between TPR domains and PUF domains, such as those found in FBF-1 and FBF-2. PUF and TPR containing proteins are members of a larger family of helical repeat proteins, characterized by the presence of an extended surface thought to be important for both protein-protein interactions and RNA binding [40]. Thus, structural similarities between TPR containing proteins such as TRD-1 and PUF containing protein such as FBF might indicate common targets, such as FEM-3.

Similar to Mog genes, *trd-1(RNAi)* animals also have defects in the mitosis to meiosis switch, with Mog genes and *trd-1(RNAi)* animals all displaying a Tum phenotype only in the sensitized background of a *gld-3* mutant [31], [38]. This is not a general characteristic of the masculinized germline, as it does not occur in a *fem-3(gf)* background [31]. It is intriguing that *trd-1(RNAi)* animals display alterations in the morphology of cytonemes, even though the mitotic region remains unchanged when *trd-1* function alone is compromised. Perhaps this is not so surprising, as the size of the DTC and associated cytonemes has not been correlated to the size of the mitotic zone in non-Notch mutant animals [6], [8]. Thus the function of the plexus (and the role of *trd-1* in influencing its morphology) remains enigmatic, although a possible role in gonadal migration has recently been postulated [8].

TRD-1 therefore has diverse roles in development, with at least three of them (seam vs. hypodermal differentiation, mitosis vs. meiosis and sperm vs. oocyte), centering on cell fate decisions. The most informative genetics analysis is performed on the best characterized genetic pathways, thus epistasis analysis using well characterized loss- and, particularly, gain-of-function alleles controlling the germ line sex determination pathway has enabled us to conclude that *trd-1* likely acts on *fem-3.* It is unknown at present what the targets of TRD-1 may be in the other developmental pathways it regulates, and the identification of genes or proteins that interact, physically or genetically, with TRD-1 will shed light on this. *trd-1* provides an additional link between the mitosis-meiosis and sperm-oocyte decisions. Thus, it is likely that *trd-1*, in common with other factors, was recruited to the germline sex determination pathway while retaining a role in germline stem cell maintenance, thus two very distinct pathways have evolved in the same tissue to control the balance between proliferation and differentiation [11]. This is intriguing in light of the role of *trd-1* in the stem-like seam lineage, where it also regulates the decision between proliferation and differentiation. It remains to be seen whether other germline cell fate regulators also have a role in seam cell development.

## Supporting Information

S1 Figure ***trd-1*** **(RNAi) animals display male tail abnormalities and molting defects.** (A) Wild type animals have 9 rays per side (strain, *N2*, *n* = 36), which is reduced to approximately 4 in *trd-1(RNAi*) animals (*n* = 75). Error bars are s.e.m. and ** indicates the 2-sample t-test where the number of rays in *trd-1(RNAi)* animals was compared to wild type, with *p*<0.01. (B) (i) Representative image of a wild type male tail where the 9 sensory rays are clearly observed. (ii) The male tails of *trd-1(RNAi)* animals display various abnormalities, including missing or fused rays and swollen bursas. (C) Molting defects in *trd-1(RNAi)* animals. Failure to shed the cuticle after molts is evident at both the anterior and posterior of the worm in around 10% of animals. Scale bar, 10 µm.
(TIF)

S2 Figure ***trd-1*** **(** ***RNAi*** **) animals have elevated sperm levels.** (A) Western blot to show sperm levels in staged young adult (L4+1 day) animals, quantified with the Major Sperm Protein (MSP) antibody 4A5. Top panel: actin loading control (band size 42 kDa). Bottom panel: MSP levels (band size 14 kDa) in wild type animals (lanes 3 and 4), feminized *fog-1(q253, lf)* mutants (lane 1), masculinized *fem-3(q20, gf)* mutants (lane 2) and masculinized *trd-1(RNAi)* animals (lane 5). *trd-1(RNAi*) animals have elevated sperm levels, similar to the *fem-3(gf)* mutant. (B) Graph to show the relative intensities of MSP levels, using ImageJ software. Wild type animals have a relative intensity of 1. *trd-1(RNAi*) animals have an elevated level of MSP, but not to the same extent as the masculinized mutant, *fem-3(gf*).
(EPS)

S3 Figure **The distal tip cell and LEPs/cytonemes have altered morphology throughout development in** ***trd-1(RNAi*** **) animals.** (A) Adult distal tip cells are formed of a plexus (comprising a cap and short intercalating processes) and long extending processes (LEPs or cytonemes) which project into the gonad. In *trd-1(RNAi*) animals, the cytonemes are thicker and extend further into the proximal gonad compared to wild type. Images are compressed maximal Z-stack projections. Scale bar, 10 µm (B) During larval stages the DTC has a migratory role then at adulthood, the DTC ceases migration and develops long extending processes (LEPs or cytonemes). LEPs are not observed in wild type animals until early adulthood (left panel). In contrast, *trd-1(RNAi*) animals (right panel) display altered DTC morphology at early stages of development. The DTC plexus appears enlarged and the LEPs form much earlier compared to wild type animals, indeed some cytonemes are present at the L2 stage. Images are compressed maximal Z-stack projections. Scale bar, 5 µm.
(TIF)
