# Supplementary figures and images for: The C. elegans TPR Containing Protein, TRD-1, Regulates Cell Fate Choice in the Developing Germ Line and Epidermis
Source: PLoS One. 2014 Dec 10;9(12):e114998. doi: 10.1371/journal.pone.0114998 (PMC4262444; doi:10.1371/journal.pone.0114998)

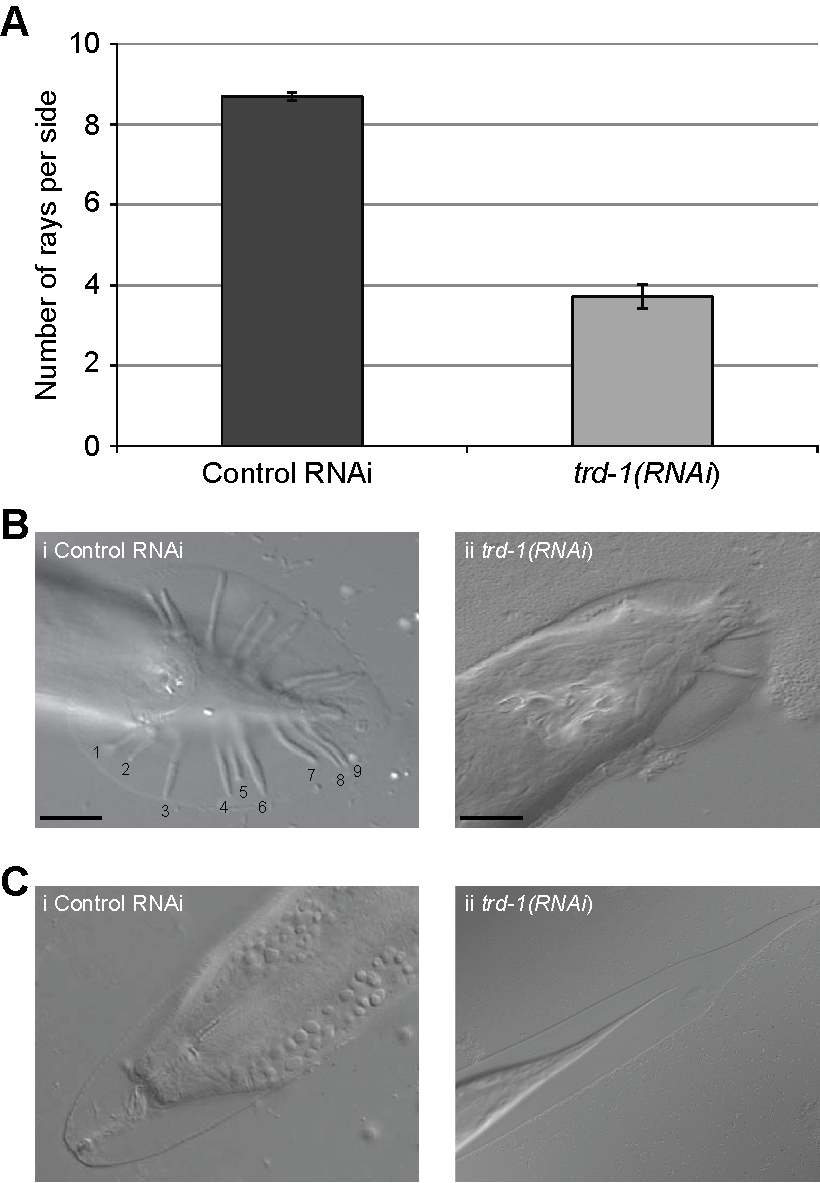

Supplement: S1 Figure — trd-1 (RNAi) animals display male tail abnormalities and molting defects. (A) Wild type animals have 9 rays per side (strain, N2, n = 36), which is reduced to approximately 4 in trd-1(RNAi) animals (n = 75). Error bars are s.e.m. and ** indicates the 2-sample t-test where the number of rays in trd-1(RNAi) animals was compared to wild type, with p<0.01. (B) (i) Representative image of a wild type male tail where the 9 sensory rays are clearly observed. (ii) The male tails of trd-1(RNAi) animals display various abnormalities, including missing or fused rays and swollen bursas. (C) Molting defects in trd-1(RNAi) animals. Failure to shed the cuticle after molts is evident at both the anterior and posterior of the worm in around 10% of animals. Scale bar, 10 µm. (TIF) [file pone.0114998.s001.tif]

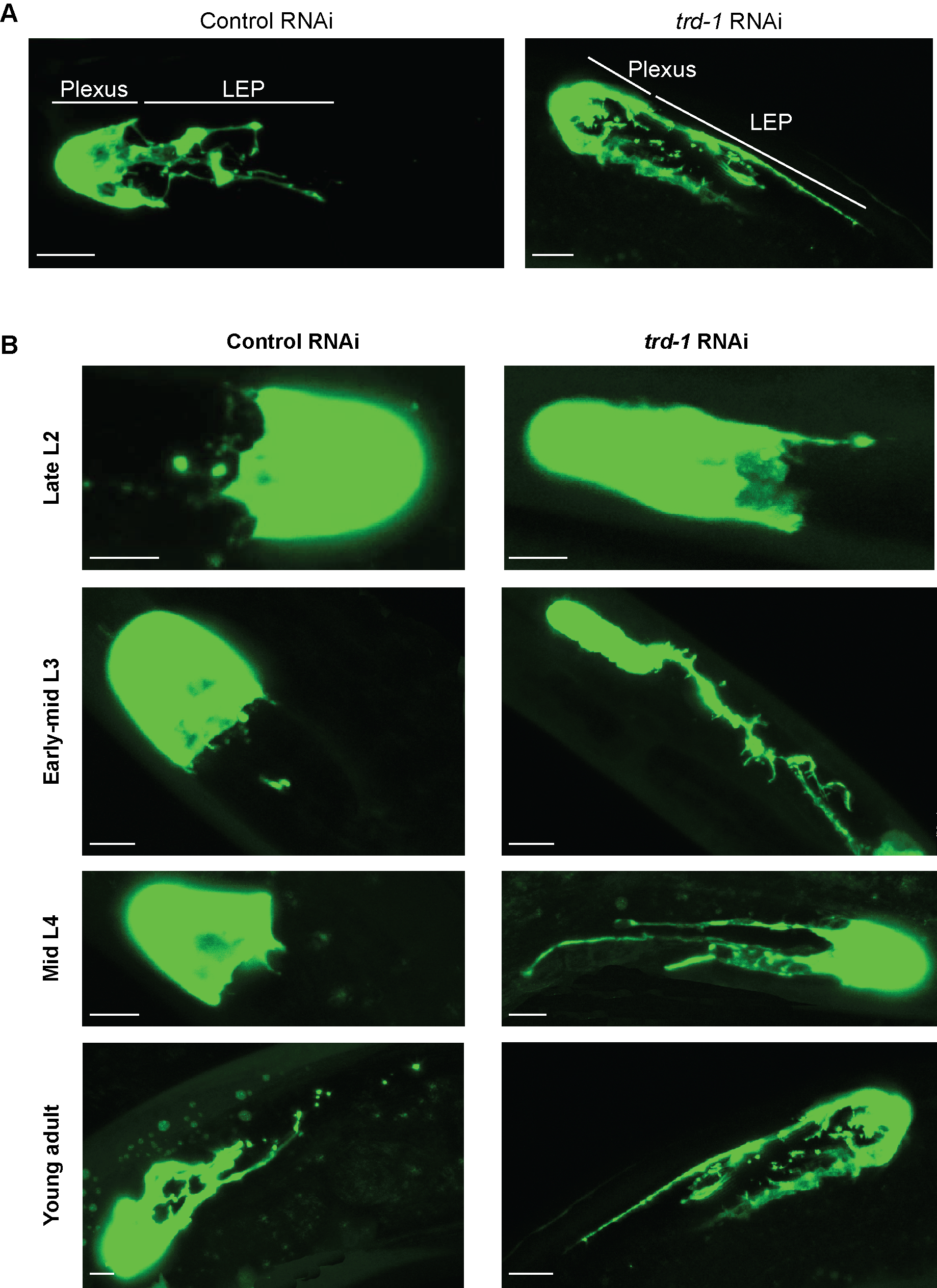

Supplement: S3 Figure — The distal tip cell and LEPs/cytonemes have altered morphology throughout development in trd-1(RNAi ) animals. (A) Adult distal tip cells are formed of a plexus (comprising a cap and short intercalating processes) and long extending processes (LEPs or cytonemes) which project into the gonad. In trd-1(RNAi) animals, the cytonemes are thicker and extend further into the proximal gonad compared to wild type. Images are compressed maximal Z-stack projections. Scale bar, 10 µm (B) During larval stages the DTC has a migratory role then at adulthood, the DTC ceases migration and develops long extending processes (LEPs or cytonemes). LEPs are not observed in wild type animals until early adulthood (left panel). In contrast, trd-1(RNAi) animals (right panel) display altered DTC morphology at early stages of development. The DTC plexus appears enlarged and the LEPs form much earlier compared to wild type animals, indeed some cytonemes are present at the L2 stage. Images are compressed maximal Z-stack projections. Scale bar, 5 µm. (TIF) [file pone.0114998.s003.tif]
